# Learning from high risk industries may not be straightforward: a qualitative study of the hierarchy of risk controls approach in healthcare

**DOI:** 10.1093/intqhc/mzx163

**Published:** 2017-12-27

**Authors:** Elisa G Liberati, Mohammad Farhad Peerally, Mary Dixon-Woods

**Affiliations:** 1 THIS Institute (The Healthcare Improvement Studies Institute), University of Cambridge, Cambridge Biomedical Campus, Clifford Allbutt Building, Cambridge CB2 OAH, UK; 2 Department of Health Sciences, Social Science Applied to Healthcare Improvement Research (SAPPHIRE) Group, University of Leicester, Leicester, UK

**Keywords:** risk control, patient safety, hierarchy of control, high reliability

## Abstract

**Objective:**

Though healthcare is often exhorted to learn from ‘high-reliability’ industries, adopting tools and techniques from those sectors may not be straightforward. We sought to examine the hierarchies of risk controls approach, used in high-risk industries to rank interventions according to supposed effectiveness in reducing risk, and widely advocated as appropriate for healthcare.

**Design:**

Classification of risk controls proposed by clinical teams following proactive detection of hazards in their clinical systems. Classification was based on a widely used hierarchy of controls developed by the US National Institute for Occupational Safety and Health (NIOSH).

**Setting and participants:**

A range of clinical settings in four English NHS hospitals.

**Results:**

The four clinical teams in our study planned a total of 42 risk controls aimed at addressing safety hazards. Most (*n* = 35) could be classed as administrative controls, thus qualifying among the weakest type of interventions according to the HoC approach. Six risk controls qualified as ‘engineering’ controls, i.e. the intermediate level of the hierarchy. Only risk control qualified as ‘substitution’, classified as the strongest type of intervention by the HoC.

**Conclusions:**

Many risk controls introduced by clinical teams may cluster towards the apparently weaker end of an established hierarchy of controls. Less clear is whether the HoC approach as currently formulated is useful for the specifics of healthcare. Valuable opportunities for safety improvement may be lost if inappropriate hierarchical models are used to guide the selection of patient safety improvement interventions. Though learning from other industries may be useful, caution is needed.

## Introduction

Health systems globally continue to experience high human burden [1] and economic costs [2] associated adverse events, yet the search for improved safety has remained elusive. Healthcare is increasingly exhorted to learn from industries, such as aviation and nuclear energy, that have achieved high reliability despite operating in hazardous contexts [3–5]. As a result, tools and procedures used in other sectors are now often deployed in healthcare settings, including a range of techniques to identify hazards and risks. Examples of approaches adapted from other industries include root cause analysis of safety incidents, which is now widely used in healthcare [6]. Proactive structured risk assessment tools (such as failure modes and effects analysis) are now also found, though less commonly [7, 8].

Once hazards have been identified, of course, organizations need to introduce risk controls to mitigate or eliminate risk of harm. In the safety literature, the concept of a hierarchy of risk controls (HoC) has gained in popularity [9–11]. The defining characteristic of the HoC approach is that it ranks risks controls according to the presumed degree of effectiveness in reducing risk [9–11], thus seeking to inform optimal choice of safety improvement strategies. Most variants of the HoC are based on three basic principles [10–13]. The first is that safety incidents occur as a result of exposure to particular hazards, implying that risk controls that eliminate the underlying hazard(s) are most likely to be effective [11, 12]. Second, HoCs are based on the assumption that humans are fallible. Accordingly, risk controls that rely on ‘hard stops’ and forcing functions—and thus minimize reliance on human behaviour—are seen as more effective on grounds that they reduce the chance for human error [13]. Third, the HoC approach is founded in the assumption that risk controls that are higher in the hierarchy are likely to be harder, on average, to design and implement [12, 13]. HoCs characteristically classify instructional or administrative solutions (like training or rewording of policies) as weak because they are thought to address only the symptoms of more institutionally engrained problems rather than the true causes.

**Figure 1:**
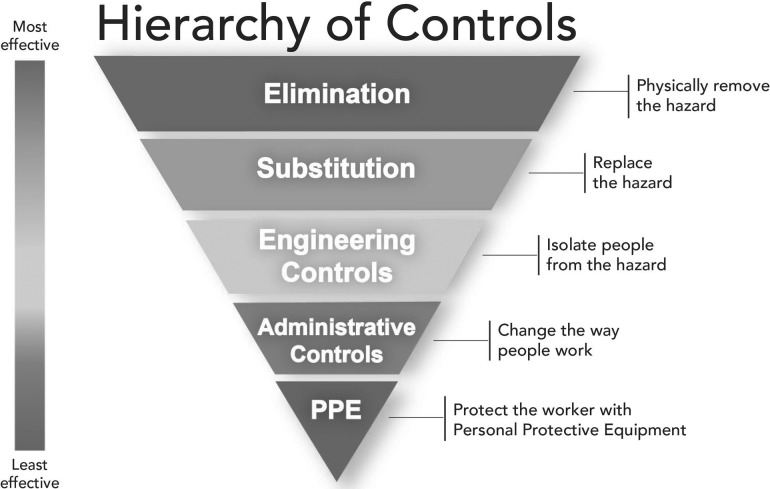
Hierarchy of control. Source: National Institute for Occupational Safety and Health (NIOSH), Division of Applied Research and Technology.

The HoC approach has, like other techniques from high-risk industries, been widely advocated in healthcare. The National Institute for Occupational Safety and Health (NIOSH) HoC [13] (Fig. 1) has been widely adopted and influential in health systems globally [14–20]. Consistent with the basic principles underlying HoCs, the NIOSH model ranks risk controls such that, e.g. product redesign is considered a stronger and more desired action than training.

When applied in a healthcare context, the available literature indicates that administrative controls, which are ranked among the weakest by NIOSH and other HoCs, remain among the most commonly proposed solutions to hazards [21, 22]. For example, a recent systematic review that used a simplified version of the NIOSH hierarchy [11] to classify risk controls in studies of root cause analysis in healthcare found that only 3.3% could be classified as elimination measures (strong) and that most (78%) of controls were administrative in nature. This review concluded by expressing concern that some of the most widely used risk control strategies in healthcare, such as training and education, ‘might do more harm than good’ [11].

Most studies to date have been conducted in the context of root cause analyses of known incidents; it is possible that more proactive approaches to hazard and risk detection might stimulate a different range of risk controls. Examination of risk controls introduced by clinical teams following proactive risk detection affords an opportunity to revisit the HoC approach and reflect on its appropriateness for healthcare, and is the task to which this article is addressed.

## Methods

We examined 42 risk controls that were planned by clinical teams in four NHS hospitals in England and Scotland. All of the teams had been trained in and were using an approach known as Safer Clinical Systems, between 2014 and 2016.

The Safer Clinical Systems approach was developed by a team at the University of Warwick with funding from the Health Foundation, an independent charitable foundation. The approach aims to support the delivery of safe and reliable healthcare based on learning from a range of hazardous industries and literatures, including high reliability organizations, risk management techniques and quality improvement methods. Safer Clinical Systems involves applying a specific set of tools and techniques—such as Failure Modes and Effects Analysis and Hierarchical Task Analysis—to local clinical systems and pathways to proactively diagnose hazards and then implementing risk control interventions to address these hazards. The approach involves four steps: (i) pathway definition and context; (ii) system diagnosis; (iii) option appraisal and planning interventions/risk controls and (iv) system improvement cycles involving implementation, revision of interventions and measurement of outcomes. A programme to test and develop the Safer Clinical Systems approach was conducted between 2011 and 2016, working with hospital sites around the UK. The Health Foundation sponsored an independent evaluation of the programme [23], on which this study is based.

Each site participating in the programme appointed a multidisciplinary team of clinicians with protected time for the programme. Between 2014 and 2016, a period known as the extension phase, the programme involved six sites and focused on understanding the extent to which clinical teams were using the Safer Clinical Systems approach to develop effective risk controls. Sites were expected to develop a set of risk controls and generate an implementation plan based on the previous diagnostic phases. Of the six sites, four implemented a set of risk controls as part of their Safer Clinical Systems activities and are the focus of our study. The remaining two sites did not progress to developing risk controls during the programme.

Of the four sites that implemented risk controls, two aimed to improve the prompt recognition and treatment of two high-risk conditions (sepsis and venous thromboembolism (VTE), respectively). The two other sites focused on improving medication safety. All teams included doctors and nurses from different medical specialties; two also included pharmacists. Appendix 1 provides a summary of the diagnostic activities undertaken by each site, the identified hazards and the risk controls (interventions aimed at improving safety) that were implemented to address these hazards.

We classified the safety interventions implemented within each of the four projects according to the five-tiered HoC proposed by NIOSH [13]. Two authors (E.L. and M.F.P.) independently assigned the interventions to the HoC, achieving 85.7% agreement on the categorization. Discrepancies were settled through discussion and the third author (M.D.W.), reaching 100% consensus on the categorization.

## Results

**Figure 2:**
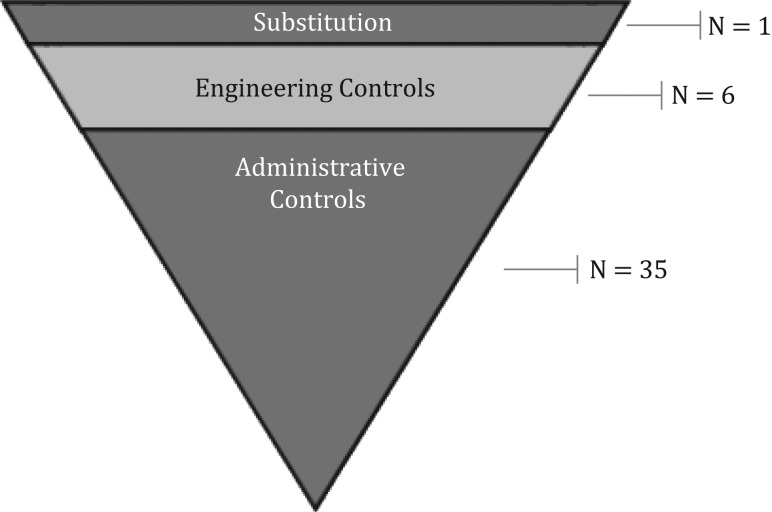
Distribution of interventions classified according to the NIOSH hierarchy of risk controls.

Between them, the four Safer Clinical Systems teams proposed 42 risk controls, which they developed following diagnosis of hazards along their respective clinical pathways. Table 1 organizes the risk controls into common themes and classifies them according to the NIOSH hierarchy of control. As shown in Fig. 2, the majority of risk controls clustered at the bottom of the hierarchy.

**Table 1:** Description and categorization of the 42 risk controls proposed by four Safer Clinical Systems teams

Category	Examples	Number	HoC category
Introduction of new equipment to substitute inappropriate/hazardous ones	Substitution of the pumps for venous thromboembolism (VTE) mechanical prophylaxis	1	1 Substitution (Strong action)
Introduction of new pieces of technology to improve complex working activities	Introduction of an electronic system for medicine reconciliation	5	6 Engineering control (intermediate action)
Forcing functions	Introduction of a computerized prescribing software that required a pharmacist to check a prescription before it was released	1	
Changes in the design and/or organization of ward care	Creation of a ‘sepsis trolley’ to ease clinicians’ access to relevant equipment Reduction of noise and disturbance during key processes	10	35 Administrative control (weak action)
Formalizing roles and responsibilities, effective use of skill mix, restructuring working shifts	Introduction of senior nursing support on the shop floor Introduction of ward champions	10	
Standardization of key processes and procedures	Standardization of VTE risk assessment according to NICE guidelines	3	
Improvement of communication via structured handovers and formal meetings	Implementation of a twice daily ‘pre-brief’ to improve multidisciplinary communication	3	
Training	Training for all nursing staff on the presentations, screening tools, and atypical presentations of sepsis	3	
Cross-checking and safety monitoring	Introduction of a pharmacist on the ward to check medicine reconciliation and, on finding a discrepancy, instructs the person responsible and talks him/her through the error	2	
Patients’ input	Patient survey on their perception of medicine reconciliation	2	
Rewards systems for best performances	Introduction of a poster or noticeboard that shows, monthly, which employer performed best in selected safety-critical tasks	1	
Others		1	
		Total 42	

Only one of the risk controls we examined—new pumps for VTE mechanical prophylaxis—qualified as ‘substitution’ (among the strongest type of risk control, according to the NIOSH hierarchy). The diagnostic work at the site where this risk control was introduced had revealed that three different forms of mechanical prophylaxis were in use in different parts of the hospital, and the chosen risk control was to replace old devices with a new, standardized one.

Six risk controls qualified as ‘engineering’ controls (the intermediate level of the HoC). An example was prescribing software that required a pharmacist to check a prescription before it was released.

The vast majority of the interventions (35 out of 42) qualified as administrative controls and would thus be deemed ‘weak’ according to the NIOSH hierarchy. This category included a highly heterogeneous group of safety actions. Ten interventions were changes in the organization and delivery of ward-based care, such as the introduction of new ways of organizing medical notes. Ten others included actions aimed at formalizing roles and responsibilities and improving the use of skill mix. These included, for example, introducing ward champions or increasing the number of hours a pharmacist could spend on a ward. Three interventions were based on training. Finally, three aimed at improving communication via structured handovers and formal meetings.

## Discussion

Our study suggests that if a hierarchy of control model adapted from high reliability industries is applied to risk controls introduced by clinical teams in response to proactive identification of hazards in their clinical pathways, most risk controls would be deemed ‘weak’ [9]. These findings are largely consistent with previous analyses of risk controls implemented following retrospective root cause analyses, suggesting that a proactive hazard detection approach does not result in a distinctive pattern of risk controls [11]. While one interpretation is that healthcare organizations are simply very poor at generating risk controls, these findings raise the question of whether a hierarchical approach to risk controls is appropriate for the specifics of healthcare. We suggest that the ability of the HoC approach to predict the success or failure of risk controls in clinical settings is challenged by three issues.

First, the HoC approach tends to categorize interventions based only on superficial and visible characteristics, without sufficient attention to the heterogeneity of risk controls and the quality of the design, delivery and intervention. For example, ‘training’ features as a self-contained category in the HoC and is classed a weak action. Yet education, training and behaviour change interventions can be delivered in multiple different formats, ranging from didactic approaches to immersive group-based simulations: they are not one thing, nor are their impacts equal [24]. Training is a key feature of some important and successful healthcare improvement programmes [25]. Training outcomes can span from enhancing technical knowledge to improving motivation, nurturing a safety culture, or developing new communities of practice [24, 26, 27]. Describing training merely as an action at the level of an ‘administrative control’ is thus reductive and misleading [28, 29]. More broadly, some risk controls deemed ‘weak’ under a HoC model (e.g. the redesign of teams or the introduction of structured communication strategies) are sometimes shown to be highly effective when they are targeted to specific groups of clinical staff, trigger experiential learning and exploit the ‘natural networks’ of healthcare [24, 30]. Valuable opportunities for tackling persistent challenges to healthcare safety, such as poor teamwork and communication between different disciplines [31], may be lost if healthcare contexts were to discard these interventions.

Second, HoCs do not take into account the fit between the identified hazards and the planned interventions, thus tending to assume that interventions will operate in the same way regardless of context. Insufficient attention is granted by the HoC approach to the degree of ‘congruence’ between a risk control and its target. This is problematic, because risk controls that rely heavily on human behaviours may feature lower in the HoC hierarchy, but may be highly impactful if designed to be congruent with the identified risks and implemented with a rigorous theory-of-change [32–34].

Third, a hierarchy-based approach may have little to offer to our understanding of the social and organizational factors that contribute to the success or failure of safety interventions in healthcare. Risk controls are created by two intertwined elements: the ‘content’ of the interventions (the core corrective action that is thought to reduce risk) and the supportive/facilitative factors that make it possible for the intervention to be implemented in specific organizational contexts [35]. Such factors include high-level organizational and managerial sponsorship, teams’ ability to engage influential clinicians, and the professional legitimization of the proposed risk controls. HoCs only account for the content element of risk controls: they do not describe how interventions are carried out nor how they become embedded and sustained over time. Moreover, since the HoC tends to conceptualize risk controls in a direct and mechanistic way, it may fail to capture the non-linear, indirect and longer-term outcomes of ‘weaker’ forms of risk controls, such as cultural change [36].

Though some features of the hierarchy of controls approach may be relevant to healthcare (e.g. its use as a structured brainstorming technique [37]), we propose that the straightforward application of this model adds little value to the development of effective risk controls in clinical settings, and lacks validity and usefulness. A better option may be to adopt models that categorize risk controls but do not imply a hierarchy. Vincent and Amalberti [26], e.g. have identified a typology of risk controls that includes five safety strategies, each of which includes different interventions. This model is not hierarchical; it does not imply that interventions can be strong or weak per se. Rather, the effectiveness of a strategy depends on the fit (‘congruence’) between the features of the local contexts and the interventions put in place. For example, in settings where care can be precisely defined, delineated strategies to control exposure to risk and maintain standards are the most suitable and promising approaches. In contrast, in more fluid and dynamic environment, strategies to improve monitoring and adaptation may be preferred.

This approach suggests that evaluation of risk controls should include assessment of the congruence between hazards and interventions, and the process and mechanisms through which interventions are expected to accomplish the desired outcome. It also suggests a need to ensure that the interventions are both comprehensive and specific enough to target the identified risks [32]; this is essential to secure the cost-effectiveness of the selected risk controls, i.e. to avoid implementing costly and complex interventions when cheaper and simpler options are available and equally effective [2]. A sound link between hazards and risk controls—i.e. a clearly outlined theory of change—is key for successful safety intervention [24, 32, 33]. In the absence of a theoretical explanation of the mechanisms through which they address specific hazards, it makes little sense to make a priori judgements about the effectiveness of a risk control and a hazard.

Some existing studies are making promising moves in this direction. Manuele [9], for instance, has suggested integrating the HoC into a structured decision-making strategy. If, e.g. a hazard is caused by individuals lacking knowledge of specific clinical procedure, then training and education operating at the individual-/team-level may be more effective than engineering controls operating at the infrastructure level. Similarly Card *et al.* [37] suggest using HoCs as a cognitive aid to supporting the formulation of risk controls. The authors propose a structured brainstorming technique (Generating Options for Active Risk Control—GO-ARC) in which a series of prompts are used to elicit risk control options after a formal risk assessment. Each prompt overlaps with a risk control strategy. This tool was shown to improve the quantity, and variety of risk control options generated. Further evaluation of such tools is needed.

## Conclusion

Assessment of risk controls should be based on the findings of empirical research and good theory about how to make improvements in healthcare, rather than rigid adoption of a hierarchical approach borrowed from other industries. The complexity of healthcare organizations, and their inherent reliance on human behaviour, interactions and knowledge, indicates that the mere duplication of ideas from other fields—even when they are deemed ‘high reliability’—may turn out to be ineffective, if not harmful. Rigid or unreflective application of the HoC approach in healthcare could mislead those seeking to improve patient safety. Introducing new tools and procedures should not be conceived in isolation from healthcare’s broader organizational context and unique features [38, 39]; achieving high reliability is more likely to occur if the adoption (and adaptation) of tools from other industries is combined with deep insights into the specific context of healthcare [39].

## Supplementary Material

Supplementary Data

Supplementary Data
