# Supplementary material for: Learning from high risk industries may not be straightforward: a qualitative study of the hierarchy of risk controls approach in healthcare
Source: Int J Qual Health Care. 2017 Dec 27;30(1):39–43. doi: 10.1093/intqhc/mzx163 (PMC5890869; doi:10.1093/intqhc/mzx163)
Supplement: Supplementary Data [file figure1permission.docx]

Elisa Liberati [egl24@medschl.cam.ac.uk]



To:

Mary Dixon-Woods ‎[md753@medschl.cam.ac.uk]‎

Cc:

Peerally, Mohammad F. (Dr.) ‎[mfp6@leicester.ac.uk]‎

Categories:

Hi Mary and Farhad,

We have received permission from NIOSH to use the attached image - please see e-mail below.

With best wishes

Elisa

Dr Elisa Liberati

[egl24@medschl.cam.ac.uk](https://owauk.oup.com/owa/ijqhc.editorialoffice@oup.com/redir.aspx?C=GyuY9tjMnbAC3DlcQgFO91Wue0KZKP4RURWYkQ_Y_Y54OS4u1y3VCA..&URL=mailto%3aegl24%40medschl.cam.ac.uk) | +44 (0)1223 76157

_____________________________
From: McCleery, Trudi (CDC/NIOSH/DART) <[taz0@cdc.gov](https://owauk.oup.com/owa/ijqhc.editorialoffice@oup.com/redir.aspx?C=vhWnNuRge1NbUWuy-9jLt-ZmexIYH6jHPzxI3E24vch4OS4u1y3VCA..&URL=mailto%3ataz0%40cdc.gov)>
Sent: Thursday, November 16, 2017 8:08 pm
Subject: your recent inquiry _ NIOSH Hierarchy of Controls
To: Elisa Liberati <[egl24@medschl.cam.ac.uk](https://owauk.oup.com/owa/ijqhc.editorialoffice@oup.com/redir.aspx?C=GyuY9tjMnbAC3DlcQgFO91Wue0KZKP4RURWYkQ_Y_Y54OS4u1y3VCA..&URL=mailto%3aegl24%40medschl.cam.ac.uk)>
Cc: Sherman, Carla D. (CDC/NIOSH/EID) <[cds4@cdc.gov](https://owauk.oup.com/owa/ijqhc.editorialoffice@oup.com/redir.aspx?C=7Nrw0WTxooNPNKLwdOUhd_ydULqUKvZIFMbE8KPscep4OS4u1y3VCA..&URL=mailto%3acds4%40cdc.gov)>, Hall, Ronald M. (CDC/NIOSH/DART) <[rmh2@cdc.gov](https://owauk.oup.com/owa/ijqhc.editorialoffice@oup.com/redir.aspx?C=ym2Ha0FFw49aO4IHA21vcog2hyjEWGnJZR5LSlYEZDJ4OS4u1y3VCA..&URL=mailto%3armh2%40cdc.gov)>

Dear Elisa,

Thank you for your recent inquiry. NIOSH gives you permission to reproduce the Hierarchy of Controls image. We appreciate you using it in your publication and citing us as the source. It is attached for your use!

If you have any additional questions or needs, please let me know.

**Trudi McCleery, MPH**

**Health Communications Specialist**

Centers for Disease Control and Prevention
National Institute for Occupational Safety and Health
Division of Applied Research and Technology
Engineering and Physical Hazards Branch
**1090 Tusculum Ave, MS R-5**
**Cincinnati, Ohio  45226**

**513-841-4591**

[**Taz0@cdc.gov**](https://owauk.oup.com/owa/ijqhc.editorialoffice@oup.com/redir.aspx?C=RtkH4h8WJ7nbemNl6FnOFyuCeGvIwCrGqz8cwG1rxKJ4OS4u1y3VCA..&URL=mailto%3aTaz0%40cdc.gov)

NAME:

Elisa Liberati

TITLE:

Dr

ORGANIZATION:

University of Cambridge

TELEPHONE:

EMAIL:

[egl24@medschl.cam.ac.uk](https://owauk.oup.com/owa/ijqhc.editorialoffice@oup.com/redir.aspx?C=GyuY9tjMnbAC3DlcQgFO91Wue0KZKP4RURWYkQ_Y_Y54OS4u1y3VCA..&URL=mailto%3aegl24%40medschl.cam.ac.uk)

STATE:

none - outside US

COUNTRY:

United Kingdom

QUESTIONS:

Dear NIOSH webmaster,

My co-authors (Dr Peerally and Professor Dixon-Woods) had a paper accepted for publication on the International Journal for Quality in Health Care (IJQHC)[https://academic.oup.com/intqhc](https://owauk.oup.com/owa/ijqhc.editorialoffice@oup.com/redir.aspx?C=BXHP1tN_PFI5R38flZHf_wi0Fx1BUo9b_C5z2tfTc2V4OS4u1y3VCA..&URL=https%3a%2f%2facademic.oup.com%2fintqhc) . We would like to ask permission to reproduce the Hierarchy of Controls image we found on your website on ([https://www.cdc.gov/niosh/topics/hierarchy/default.html](https://owauk.oup.com/owa/ijqhc.editorialoffice@oup.com/redir.aspx?C=xsVaecPXjptBe9ZfQUH9iSDAWeiE8G_Oi3c5WGumIDV4OS4u1y3VCA..&URL=https%3a%2f%2fwww.cdc.gov%2fniosh%2ftopics%2fhierarchy%2fdefault.html) ) in our article.

If you are happy for us to use the image and acknowledge you as the source, would you please send us an original source file?

Many thanks indeed for your help. Please do not hesitate to contact me if you need any further information.

With best wishes

Elisa
