# Supplementary material for: Learning from high risk industries may not be straightforward: a qualitative study of the hierarchy of risk controls approach in healthcare
Source: Int J Qual Health Care. 2017 Dec 27;30(1):39–43. doi: 10.1093/intqhc/mzx163 (PMC5890869; doi:10.1093/intqhc/mzx163)
Supplement: Supplementary Data [file appendix1new222092017.docx]

Appendix 1: Diagnostic activities, identified hazards, and the safety interventions in each participating site.

| **Site** | **Diagnostic tools** | **Diagnosed hazards** | **Risk controls** |
| --- | --- | --- | --- |
| **Site 1** | - Process mapping - Failure Modes Effects Analysis (FMEA) - Hierarchical Task Analysis (HTA) - Fishbone analysis - Swim lane mapping - Collection of qualitative data from key staff, who were asked to list what they felt were the barriers to providing optimal care | - Sepsis not being recognised on initial presentation - Patients being allocated incorrect location in the department in relation to care needs - Sepsis not being identified by nursing assessment - Sepsis not being identified by medical assessment - The appropriate sepsis investigations not being completed in a timely manner - Correct treatment not being identified or prescribed - Delays in treatment delivery - Lack of accurate treatment documentation - Insufficient patient monitoring - Delay in transfer to step up/ step down care | 1. **Training for all nursing staff** on the presentations, screening tools, and atypical presentations of sepsis. 2. **Implementation of sepsis stickers** on suspicion of severe sepsis or septic shock to The provide a handy reminder of the urgent steps needed. 3. **Implementation of the sepsis trolley** to easeclinicians’ access to the equipment required to investigate, treat, and monitor septic patients. 4. **Implementation of a twice daily “pre-brief” to enhance communication** and awareness of skills, roles, and competencies among all the staff at the shop floor. 5. **Implementation of name boards to make the named nurse visible in each room** to improve communication between colleagues and enhance patient dignity and respect. 6. **Introduction of senior nursing support on the shop floor** to ensure appropriate and safe patient pathways in the busy environment of the Accident & Emergencies (A&E). |
| **Site 2** | - Process mapping - FMEA (which results were consistent with the ones from RCAs conducted prior to the beginning of the project) | - Trust policy not compliant with national guidance - No clear delegation of responsibility in place to complete the VTE risk assessment on admission - Inaccurate VTE risk assessment completed by nursing staff in pre-operative assessment unit and filed in patient’s health record - Lack of patient’s weight documented on the medication chart leading to inaccurate prescribing of pharmaceutical prophylaxis - VTE risk assessment not completed on the day of admission for elective surgical patients - Pharmaceutical VTE prophylaxis prescribed without evidence of a VTE risk assessment being completed. - No clear VTE prophylaxis instructions documented in the surgical operation note or prescribed on the drug chart - Patients arrive in theatre recovery with no VTE risk assessment completed or prophylaxis plan documented on the operation note - Mechanical prophylaxis not routinely applied by nurses on admission to ward - VTE re-risk assessment rarely completed - Lack of medical and nursing knowledge regarding VTE prophylaxis - Three different forms of mechanical prophylaxis were used in the Trust; orthopaedics, theatre and stroke favoured different forms. Thus patients needing prophylaxis were often left without proper prophylaxis for many hours (pumps might not be changed when a patient was moved to a different part of the hospital). | 1. **Introduction of the documentation of patients’** weight into the medication chart. 2. **Standardisation of prophylaxis**: It was established that the VTE prophylaxis (pharmacological and mechanical) needed be prescribed by surgeons as part of the VTE risk assessment in the admission suite, rather than by junior doctors as part of the clerking process for non-elective patients. 3. **New communication strategy**. Throughout the project, VTE-related procedures featured in trust magazines, open staff meeting presentations, World Thrombosis day, and various safety events held within the Trust. 4. Creation of **an e-learning package** for nurses and allied health professionals. 5. **Standardisation of VTE risk assessment**. It was decided that the VTE risk assessment needed to be completed by surgeons, in the surgical admission suite, on the day of admission, prior to going to the operating theatre. 6. **Introduction of a system** **that prevented patients leaving the admission suite unless the VTE risk assessment was completed**. This intervention meant that surgeons would be called back from theatre to complete the risk assessment when itappeared to be incomplete. 7. **Introduction of ward champions** to raise awareness of the use of mechanical prophylaxis and the responsibility of ward staff to ensure devices are applied correctly. 8. To improve standardisation of mechanical prophylaxis equipment, and avoid the risks associated with using three different pumps, **new equipment for mechanical prophylaxis** was introduced to replace the old ones. This was used throughout the trust. |
| **Site 3** | - Process mapping - FMEA - Human Factor Analysis | - The medical reconciliation sheet not fully completed on admission - One or more sources of medication on admission often unavailable or not used - Transcription from the medicine reconciliation sheet to Kardex often incorrect or incomplete - Reasons for starting and stopping medication not always documented on Kardex and notes, and therefore not reported on the IDL (Immediate Discharge Letter) - Kardex and notes not available for medication review, due to the lack of organisation of the notes trolley - Variability in the ways doctors conducted medicine reconciliation - Junior doctors not always informed as to when patients arrived to the ward - Incorrect or incomplete medication list resulting in inappropriate meds prescribed or appropriate meds missed or incorrectly written. | 1. **A sticker** was trialled that was intended to show whether medicine reviews had been done on admission and to document any medication changes made during admission. 2. **The Kardex locator**. Having learnt that much time was wasted for looking for Kardexes (files containing medical records) on the ward, the team developed a system to show where patients’ notes were at any given time comrpisinga laminated sheet with a little wheel on it (a ‘dial’). 3. **Re-organisation of the notes trolley** on the orthopaedic ward (using an alphabetical order rather than consultants’ names). 4. **The junior doctors’ whiteboard**. Since junior doctors were not always aware of the patients being admitted to the ward, a whiteboard was put in place in the junior doctors’ area to flag-up incoming admissions. 5. **Patient survey**. The team noticed that patients had often stopped taking medications that still appeared on their notes, and so they decided to explore why, and to gather patients’ views on the process of medicine reconciliation. 6. **Training for junior doctors.** 7. **Trialling of a computer-based medicines reconciliation system** which allows the collection of accurate information regarding the type and dosage of the medicines included in patients’ plans, as well as the reasons for stopping particular drugs. 8. The project team **encouraged attendance junior doctors to the morning ward round** and huddle (where the discharge process and handover of the patients were discussed). 9. The team considered establishing a “**quiet area”** for junior doctors to work, to limit interruptions and improve the accuracy of transcription of medications to the Kardex. 10. **Cross checking and monitoring**. The project pharmacist was on the ward for 2.5 days per week and was actively checking medicines reconciliation. On finding a discrepancy, the pharmacist finds the junior doctor responsible and talks them through the error and how it should be corrected. 11. **A poster**/**noticeboard** to show, monthly, whic**h junior doctor made the best job of their medicines reconciliation** work. 12. A **sample Kardex** for junior doctors to refer to when writing up the Kardex for hip and knee surgical patients. |
| **Site 4** | - Process mapping - FMEA - Swim-lane analysis - Some root cause analysis techniques (‘5 Whys’, ‘Lean A3 thinking’), - Fishbone analysis - PRIMO questionnaire - Review of governance information (adverse incidents and complaints). | - Pharmacy staff unable to see all patients/complete key tasks. - Key information lost through pharmacy to pharmacy communication. - Key information lost through multidisciplinary communication. - Inaccurate prescribing of preadmission medicines. - Inaccurate prescribing of new medicines. - Inaccurate prescribing early in the pathway. - All the above hazards exacerbated at the weekend. | 1. **Standardisation of pharmacy roles.** The team have attempted to standardise pharmacy work on the Emergency Assessment Units (EAU) EAU, for example, by identifying what was the best time for pharmacist to arrive and which huddles they needed to attend. 2. **Standardisation of pharmacy communication**. To improve communication between pharmacy and medical staff the team tested a morning pharmacy huddle. 3. **Pharmacist on ward round.** To further facilitate communication between pharmacists, doctors, and nurses, the team hypothesised that pharmacists could attend the ward round of the EAU and A&E. 4. **Extension of pharmacists’ time on EAU**. The team noticed that the two-hour pharmacists’ slot on the EAU in the weekend was associated with the loss of important information. It was therefore decided to extend the pharmacists’ slot to three hours. 5. **Extension of pharmacy hours on EAU.** The team planned an extension of pharmacy hours on the EAU. 6. **Additional pharmacy staffing at weekends**. The team requested that additional staff were put on the EAU at the weekend; changes were made with the whole weekend rota. 7. **Pharmacist on A&E**. The introduction of the electronic prescribing in the A&E meant that doctors were prescribing earlier in the pathway (in the A&E, rather than in the EAU). 8. **Improvement to Med Rec/ Electronic Documentation**. This intervention involved changing the whole way the pharmacists captured medicines reconciliations. 9. **Trial of prescribing pharmacist on the EAU.** 10. **Spread of on-hold function**. The ‘on-hold’ function on prescribing software required a pharmacist to check the prescription before it was released, helping to achieve 100% prescribing accuracy. 11. **Introduction of order sets**. To increase prescription accuracy, the team introduced order sets for antibiotics (and other medicines) to be prescribed to patients in particular conditions, for example, pneumonia and sepsis. 12. **Reliable ways to capture preadmission medicines**. Medical staff were asked to return all pre-admission documentation consistently to the reception staff after admission, so that it could be appropriately scanned. 13. **Use of technology to support pharmacy efficiency**. The team introduced two laptops on the EAU. 14. **Reliable process to put patients’ drugs away**. This intervention aimed to avoid the pharmacists re-ordering drugs that had already been delivered to EAU. 15. **Tracker of discharge drugs**,where nurses and doctors could monitor patients’ medication for taking home. The tracker was introduced in the discharge lounge on the EAU. 16. **Patient survey** to ensure that patients’ perceptions and experiences of medication safety were captured. |

Interpretation:

Intervention 14 was categorised as substitution

Interventions 21, 34, 36, 37, 39, 41 were categorised as engineering controls

All of the remainder were categorised as administrative controls
